# Supplementary figures and images for: Cost-effectiveness of short, oral treatment regimens for rifampicin resistant tuberculosis
Source: PLOS Glob Public Health. 2022 Dec 7;2(12):e0001337. doi: 10.1371/journal.pgph.0001337 (PMC10022130; doi:10.1371/journal.pgph.0001337)

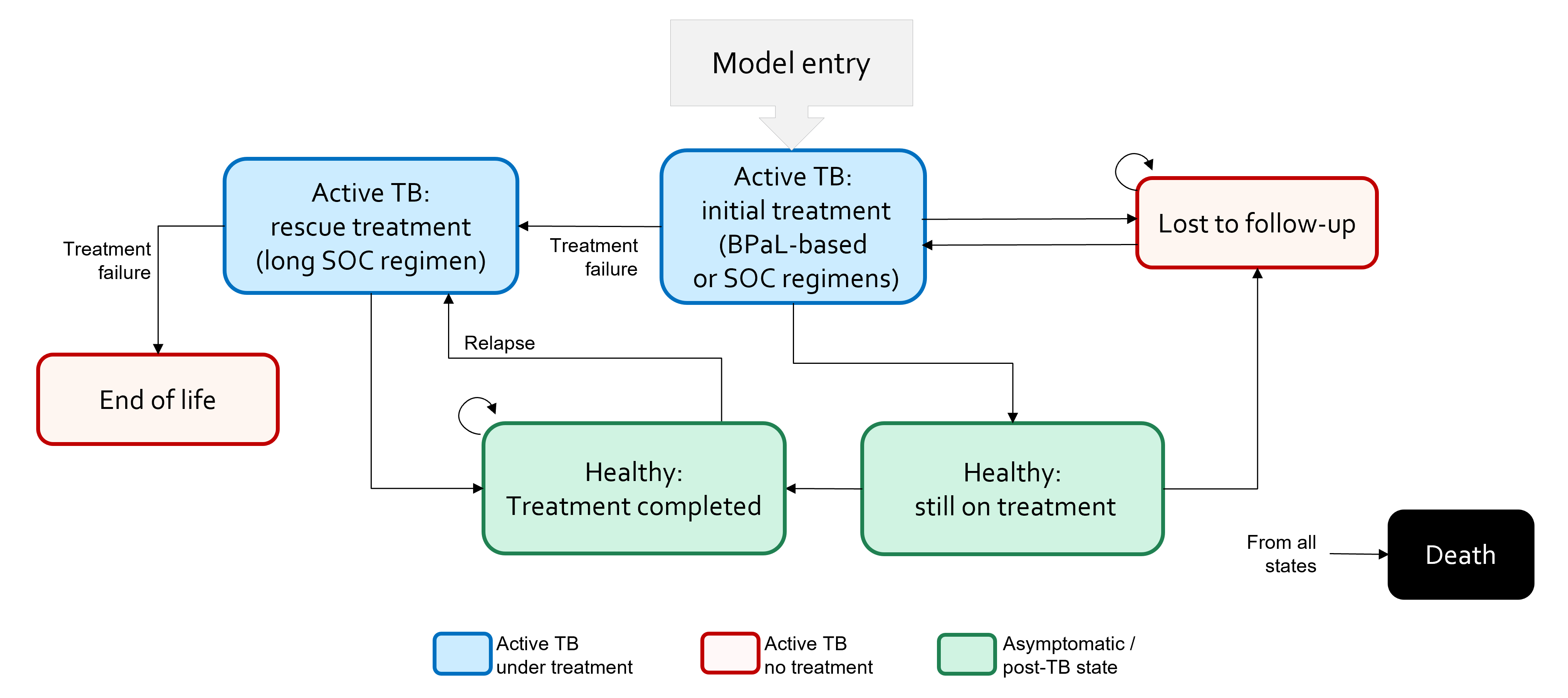

Supplement: S1 Fig — (TIF) [file pgph.0001337.s002.tif]

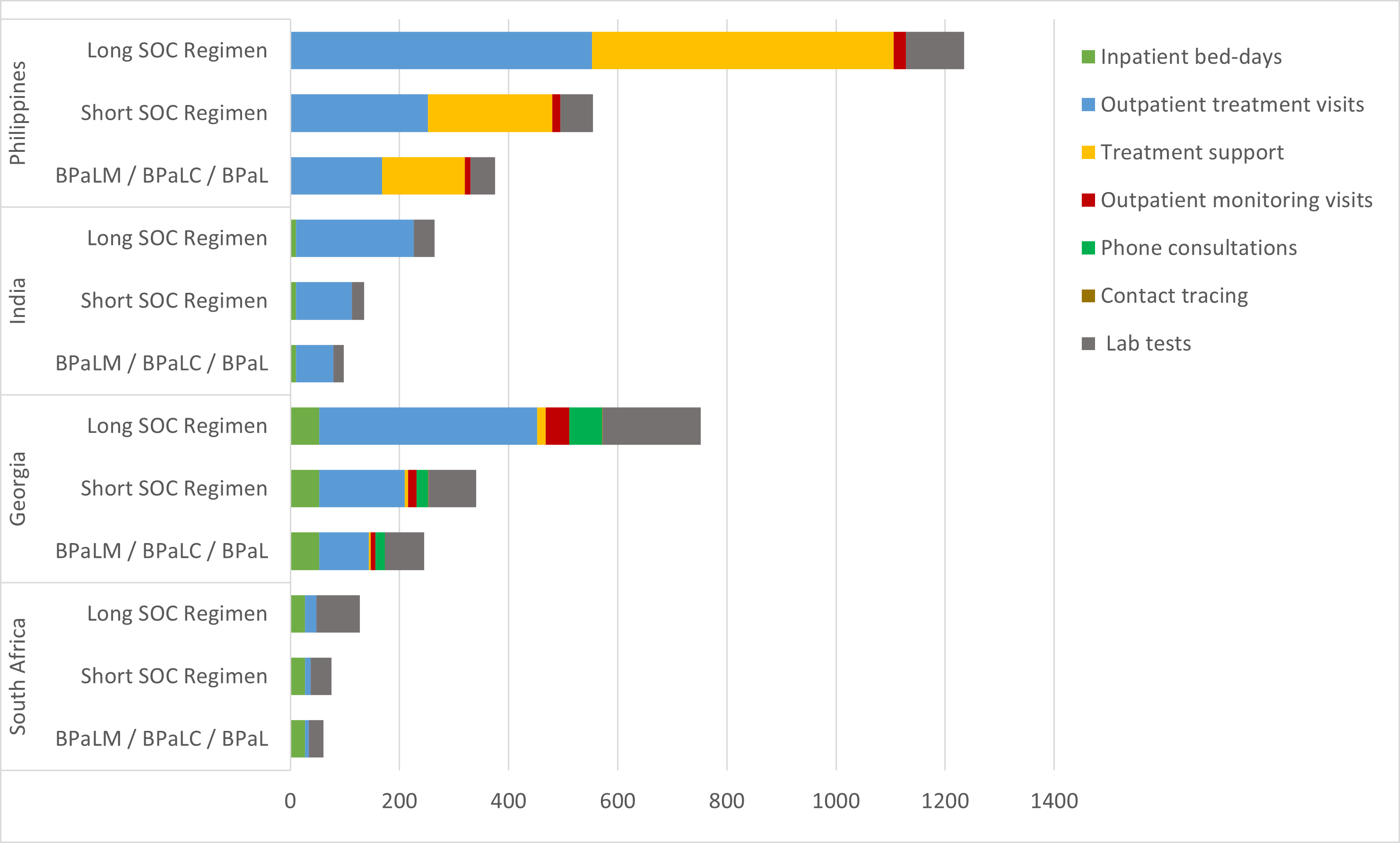

Supplement: S2 Fig — (TIF) [file pgph.0001337.s003.tif]

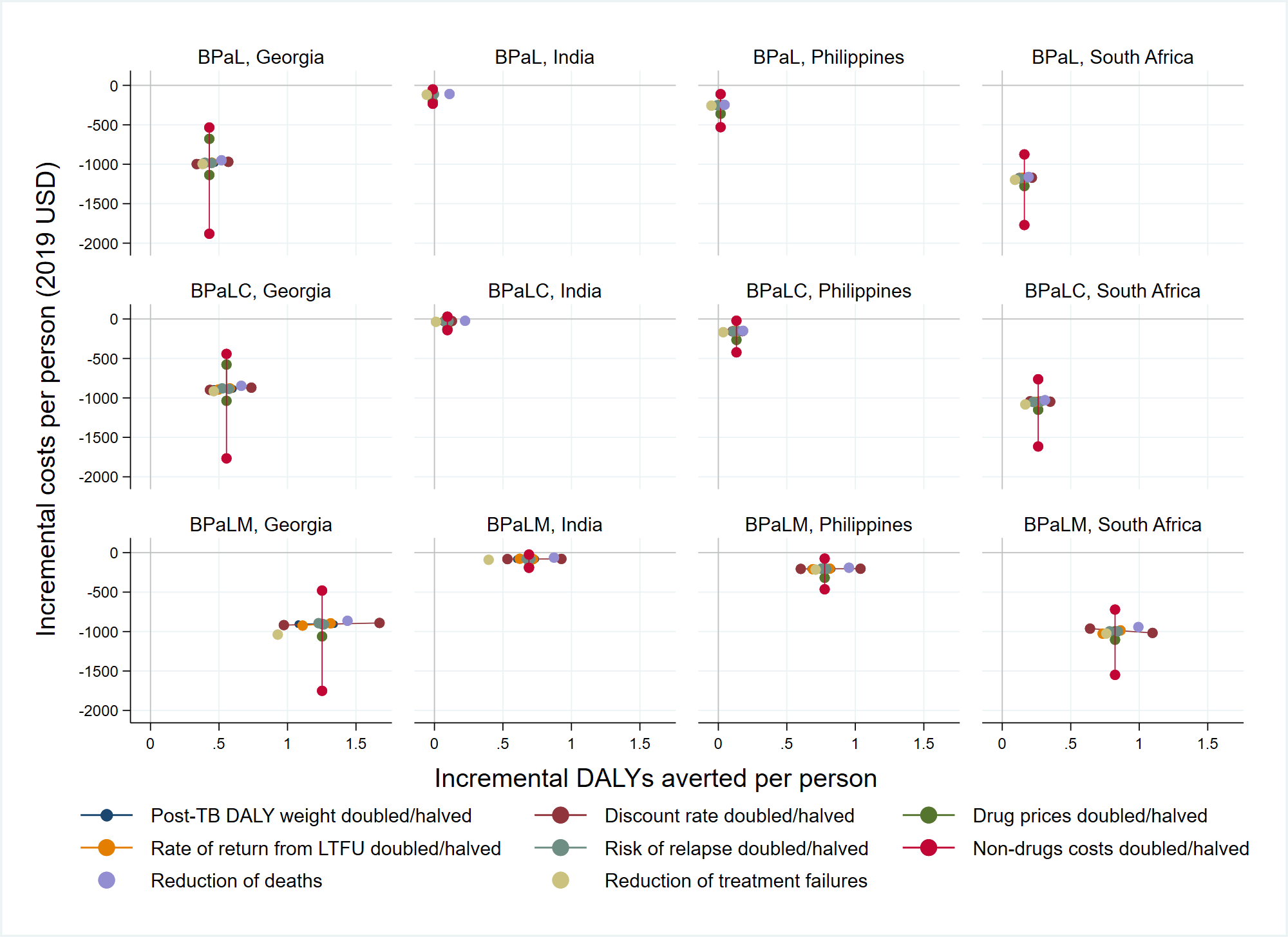

Supplement: S3 Fig — (TIF) [file pgph.0001337.s004.tif]
