# Supplementary material for: Cost-effectiveness of short, oral treatment regimens for rifampicin resistant tuberculosis
Source: PLOS Glob Public Health. 2022 Dec 7;2(12):e0001337. doi: 10.1371/journal.pgph.0001337 (PMC10022130; doi:10.1371/journal.pgph.0001337)
Supplement: S1 Table — (DOCX) [file pgph.0001337.s005.docx]

##### S1 Table. Specification of regimens

| **Drug** | **BPaLM regimen** | **BPaLC regimen** | **BPaL regimen** |
| --- | --- | --- | --- |
| Bedaquiline (Bdq) | 400 mg once daily for 2 weeks followed by 200 mg 3 times per week for 22 weeks | 400 mg once daily for 2 weeks followed by 200 mg 3 times per week for 22 weeks | 400 mg once daily for 2 weeks followed by 200 mg 3 times per week for 22 weeks |
| Pretomanid (Pa) | 200mg once daily | 200mg once daily | 200mg once daily |
| Linezolid (L) | 600mg daily for 16 weeks then 300mg daily for the remaining 8 weeks or earlier when moderately tolerated | 600mg daily for 16 weeks then 300mg daily for the remaining 8 weeks or earlier when moderately tolerated | 600mg daily for 16 weeks then 300mg daily for the remaining 8 weeks or earlier when moderately tolerated |
| Moxifloxacin (Mfx) | 400 mg once daily |  |  |
| Clofazimine (Cfz) |  | 50 mg (less than 33 kg), 100 mg (more than 33 kg) |  |
