# Supplementary material for: Cost-effectiveness of short, oral treatment regimens for rifampicin resistant tuberculosis
Source: PLOS Glob Public Health. 2022 Dec 7;2(12):e0001337. doi: 10.1371/journal.pgph.0001337 (PMC10022130; doi:10.1371/journal.pgph.0001337)
Supplement: S2 Table — (DOCX) [file pgph.0001337.s006.docx]

##### S2 Table. Model Parameters

| **Parameter** | **Value** | **SE** | **PSA Distribution** | **Reference** |
| --- | --- | --- | --- | --- |
| Average age at model start (years) | 35 |  | N/A | assumption |
| Average body weight (kilograms) | 51-70 |  | N/A | assumption |
| Discount rates for costs and effects | 3% |  | N/A | (16) |
| Risk ratio for treatment success: BPaLM | 1.10 | 0.07 | Log normal | (17) |
| Risk ratio for treatment success: BPaLC | 1.02 | 0.08 | Log normal | (17) |
| Risk ratio for treatment success: BPaL | 1.01 | 0.08 | Log normal | (17) |
| Annual risk of relapse in year 1 post-treatment | 2.80% | 0.40% | Normal | (18) |
| Annual risk of relapse in year 2 post-treatment | 1.00% | 0.30% | Normal | (18) |
| Annual risk of relapse in year 3 post-treatment | 0.40% | 0.20% | Normal | (18) |
| Annual risk of relapse in year 4 post-treatment | 0.30% | 0.20% | Normal | (18) |
| Hazard ratio for relapse among people with HIV | 2.40 |  | N/A | (19) |
| Annual likelihood of return to care after LTFU | 28% | 3% | Normal | (20) |
| Access to end of life care | 25% | 5% | Normal | assumption |
| Monthly probability of death for end-of-life care | 6.86% | 0.69% | Beta | (21) |
| Monthly probability of death following LTFU | 6.86% | 0.69% | Beta | (21) |

SE standard error; PSA probabilistic sensitivity analysis; N/A not applicable; HIV human immunodeficiency virus; LTFU loss to follow-up
