# Supplementary material for: Cost-effectiveness of short, oral treatment regimens for rifampicin resistant tuberculosis
Source: PLOS Glob Public Health. 2022 Dec 7;2(12):e0001337. doi: 10.1371/journal.pgph.0001337 (PMC10022130; doi:10.1371/journal.pgph.0001337)
Supplement: S3 Table — (DOCX) [file pgph.0001337.s007.docx]

##### S3 Table. DALY weights for all health states in the model

| **Condition** | **DALY Weight** | **Health state description** | **SE** | **PSA**  **Distribution** | **Source** |
| --- | --- | --- | --- | --- | --- |
| Post-TB | 0.053 |  |  | Beta | [15] |
| HIV, no active TB | 0.125 | Has weight loss, fatigue, and frequent infections. | 0.07 | Beta | [12] |
| Active TB, no HIV | 0.333 | Has a persistent cough and fever, is short of breath, feels weak, and has lost a lot of weight. | 0.06 | Beta | [12] |
| Active TB & HIV | 0.439 | combined disability weight | 0.02 | Beta | [12] |
| End of life | 0.540 | Has lost a lot of weight and regularly uses strong medication to avoid constant pain. The person has no appetite, feels nauseous, and needs to spend most of the day in bed. | 0.09 | Beta | [12] |
| Death | 1 |  |  | Beta | [12] |
| Liver dysfunction (grade 3 and above) | 0 | Asymptomatic | - | Beta | [10, 12] |
| Pancreatitis (grade 3 and above) | 0.114 | Has pain in the belly and feels nauseous. The person has difficulties with daily activities. | 0.02 | Beta | [10, 12] |
| Anaemia (grade 3 and above) | 0.052 | Has moderate fatigue, weakness, and shortness of breath after exercise, making daily activities more difficult. | 0.01 | Beta | [10, 12] |
| Neutropenia (grade 3 and above) | 0 | Asymptomatic | - | Beta | [10, 12] |
| QTcF prolongation (grade 3 or above) | 0 | Asymptomatic | - | Beta | [10, 12] |
| Vomiting (grade 3 and above) | 0.114 | Has pain in the belly and feels nauseous. The person has difficulties with daily activities. | 0.02 | Beta | [10, 12] |
| Renal disfunction (grade 3 and above) | 0.051 | Has fever and aches, and feels weak, which causes some difficulty with daily activities. | 0.01 | Beta | [10, 12] |

DALY disability-adjusted life year; SE standard error; PSA probabilistic sensitivity analysis; QT corrected for heart rate by Fridericia's cube root formula
