# Supplementary material for: Cost-effectiveness of short, oral treatment regimens for rifampicin resistant tuberculosis
Source: PLOS Glob Public Health. 2022 Dec 7;2(12):e0001337. doi: 10.1371/journal.pgph.0001337 (PMC10022130; doi:10.1371/journal.pgph.0001337)
Supplement: S4 Table — (DOCX) [file pgph.0001337.s008.docx]

##### S4 Table. Unit costs

| **Service** | **Georgia** | **India** | **South Africa** | **Philippines** | **PSA**  **Distribution** | **Source** |
| --- | --- | --- | --- | --- | --- | --- |
| Outpatient diagnostic visit | $3.69 ($0.70) | $1.11 ($0.21) | $12.00 ($2.26) | $1.99 ($0.41) | Gamma | (27,28) |
| Outpatient treatment support visit | $2.42 ($0.45) | $1.44 ($0.27) | $12.00 ($2.26) | $0.42 ($0.08) | Gamma | (27,28) |
| Outpatient treatment visit | $2.13 ($0.33) | $1.15 ($0.22) | $12.00 ($2.26) | $1.68 ($0.29) | Gamma | (27,28) |
| Outpatient monitoring visit | $3.59 ($0.65) | $2.81 ($0.53) |  | $2.05 ($0.36) | Gamma | (27) |
| Inpatient bed-day | $38.28 ($3.09) | $18.06 ($3.41) | $49.03 ($3.96) | $26.63 ($2.03) | Gamma | (27,28) |
| Community-level treatment visit | $1.88 ($0.12) | $0.54 ($0.10) |  |  | Gamma | (27) |
| Community-level other visit |  | $1.70 ($0.32) |  |  | Gamma | (27) |
| Lost to follow-up tracing: phone calls | $1.24 ($0.22) |  |  | $0.70 ($0.12) | Gamma | (27) |
| Lost to follow-up tracing: home visit |  |  | $9.43 ($2.57) |  | Gamma | (28) |
| Phone consultation | $0.53 ($0.10) |  |  |  | Gamma | (27) |
| Contact tracing | $0.50 ($0.30) |  |  |  | Gamma | (27) |
| Lab tests |  |  |  |  |  |  |
| Sputum collection | $1.63 ($0.24) |  |  |  | Gamma | (27) |
| Ziehl-Neelsen smear microscopy | $5.59 ($0.84) | $1.90 ($0.36) | $6.95 ($1.04) | $2.53 ($0.39) | Gamma | (27,28) |
| Solid sputum culture | $8.56 ($1.29) | $1.90 ($0.36) |  | $23.29 ($3.17) | Gamma | (27) |
| Sputum culture | $15.55 ($2.33) |  |  |  | Gamma | (27) |
| Electrocardiogram | $1.89 ($0.26) | $0.99 ($0.19) | $14.35 ($2.15) | $3.50 ($0.63) | Gamma | (27,28) |
| HIV rapid test | $2.58 ($0.28) | $1.29 ($0.24) |  | $3.11 ($0.42) | Gamma | (27) |
| Full haemogram | $4.01 ($0.50) | $0.65 ($0.12) |  | $2.69 ($0.96) | Gamma | (27) |
| Creatinine |  | $0.59 ($0.11) |  | $1.78 ($0.27) | Gamma | (27) |
| Blood sugar | $1.01 ($0.15) | $0.78 ($0.15) |  | $2.75 ($0.59) | Gamma | (27) |
| Thyroid-stimulating hormone test |  | $2.69 ($0.51) |  |  | Gamma | (27) |
| Chest Xray (film) | $4.45 ($0.44) | $1.99 ($0.37) |  | $2.75 ($0.51) | Gamma | (27) |
| Chest Xray (digital) | $2.63 ($0.84) | $2.05 ($0.39) |  | $1.97 ($0.50) | Gamma | (27) |
| Fasting blood sugar |  |  |  | $1.55 ($0.23) | Gamma | (27) |
| Liver function test | $2.54 ($0.38) | $2.69 ($0.51) |  | $2.92 ($0.33) | Gamma | (27) |
| Audiometry |  |  |  | $8.80 ($2.33) | Gamma | (27,28) |
| Visual acuity |  |  |  | $7.54 ($1.92) | Gamma | (27) |
| Potassium |  |  |  | $5.37 ($0.81) | Gamma | (27) |
| Biochemistry | $1.79 ($0.24) |  | $32.30 ($4.85) | $2.46 ($1.27) | Gamma | (27) |
| Electrolyte test |  |  |  | $0.95 ($0.14) | Gamma | (27) |
| Bloodgroup RH | $2.54 ($0.38) |  |  | $1.74 ($0.26) | Gamma | (27) |
| Blood clotting | $6.55 ($1.24) |  |  |  | Gamma | (27) |
| Light-emitting diode fluorescence microscopy (LED-FM) | $2.52 ($0.38) |  |  |  | Gamma | (27,28) |
| Magnetic resonance imaging | $2.26 ($0.34) |  |  |  | Gamma | (27) |
| Computerized Tomography (CT) scan | $10.10 ($1.52) |  |  |  | Gamma | (27) |
| Ultrasound test |  | $0.89 ($0.17) | $6.95 ($1.04) |  | Gamma | (27) |
| Other tests | $3.55 ($0.53) |  |  |  |  |  |
| Cost per month for antiretroviral therapy | $23.48 ($3.52) |  |  |  | Gamma | (33) |
| Cost per month for LTFU patients | $3.13 ($0.76) |  |  |  | Gamma | (27,28) |
| Cost per month for end of life state | $3.69 ($0.70) | $1.11 ($0.21) | $12.00 ($2.26) | $1.99 ($0.41) | Gamma | (27,28) |
| Cost per death | $2.42 ($0.45) | $1.44 ($0.27) | $12.00 ($2.26) | $0.42 ($0.08) | n/a | (34) |
| Cost after TB cure | $2.13 ($0.33) | $1.15 ($0.22) | $12.00 ($2.26) | $1.68 ($0.29) | n/a | assumption |
| Cost per month for liver dysfunction | $3.59 ($0.65) | $2.81 ($0.53) |  | $2.05 ($0.36) | Gamma | (27,29) |
| Cost per month for pancreatitis | $38.28 ($3.09) | $18.06 ($3.41) | $49.03 ($3.96) | $26.63 ($2.03) | Gamma | (27,29) |
| Cost per month for anaemia | $1.88 ($0.12) | $0.54 ($0.10) |  |  | Gamma | (27,29) |
| Cost per month for neutropenia |  | $1.70 ($0.32) |  |  | Gamma | (27,29) |
| Cost per month for QTcF prolongation | $1.24 ($0.22) |  |  | $0.70 ($0.12) | Gamma | (27,29) |
| Cost per month for vomiting |  |  | $9.43 ($2.57) |  | Gamma | (27,29) |
| Cost per month for renal disfunction | $0.53 ($0.10) |  |  |  | Gamma | (27,29) |
