# Supplementary material for: Cost-effectiveness of short, oral treatment regimens for rifampicin resistant tuberculosis
Source: PLOS Glob Public Health. 2022 Dec 7;2(12):e0001337. doi: 10.1371/journal.pgph.0001337 (PMC10022130; doi:10.1371/journal.pgph.0001337)
Supplement: S5 Table — (DOCX) [file pgph.0001337.s009.docx]

##### ****S5 Table. PSA results****

|  | Mean incremental costs per person | Percent simulations cost saving | Mean DALYs averted per person | Percent simulations averting DALYs |
| --- | --- | --- | --- | --- |
| Philippines |  |  |  |  |
| BPaL | -$245 | 100% | 0.03 | 50% |
| BPaLC | -$152 | 100% | 0.13 | 54% |
| BPaLM | -$199 | 100% | 0.78 | 89% |
| India |  |  |  |  |
| BPaL | -$111 | 99% | 0.01 | 49% |
| BPaLC | -$28 | 74% | 0.09 | 53% |
| BPaLM | -$79 | 94% | 0.70 | 88% |
| South Africa |  |  |  |  |
| BPaL | -$1,171 | 100% | 0.18 | 59% |
| BPaLC | -$1,053 | 100% | 0.26 | 62% |
| BPaLM | -$997 | 100% | 0.83 | 93% |
| Georgia |  |  |  |  |
| BPaL | -$993 | 100% | 0.45 | 71% |
| BPaLC | -$879 | 100% | 0.55 | 78% |
| BPaLM | -$901 | 100% | 1.25 | 97% |
