# Supplementary material for: Cost-effectiveness of short, oral treatment regimens for rifampicin resistant tuberculosis
Source: PLOS Glob Public Health. 2022 Dec 7;2(12):e0001337. doi: 10.1371/journal.pgph.0001337 (PMC10022130; doi:10.1371/journal.pgph.0001337)
Supplement: Attachment — Submitted filename: PRACTECAL CEA_Response to reviewers.docx [file pgph.0001337.s010.docx]

Reviewer #1:

This modelling based study indicates that BPaL-based regimens are likely to be cost-saving and more effective than the current standard of care in a range of settings and it suggests that countries should consider programmatic uptake of BPaL-based regimens. The study has many important implications especially in the context of poor success rates with the current regimen used for treating drug resistant TB forms such as MDR and XDR-TB and increasing levels of drug resistance in these study countries.

Following are some points to consider
1. At model entry, mean age was assumed to be 35 (Supplementary Table 2): Can authors provide justification for this number?

This was the average age for PRACTECAL participants. We have added some text to clarify this, and added a citation.

2. At the end of each month, patients could transition to the next month of treatment, loss to follow-up, treatment failure, or death: I am unable to understand how they can transition to failure just in next month? On what basis?

We use a Markov model to represent the movement of patients through various health states and activities throughout their lifetime. We follow the standard procedure for Markov models, in using monthly transition probabilities to represent movement through these states over time. The transition probabilities are calculated using the percentage of patients achieving each outcome (treatment success, treatment failure, death, loss to follow-up) by the end of their treatment period.

3. Following the failure of initial regimen, authors assume that patients are put on long SOC rescue regimen regardless of their initial regimen. What is the basis for this assumption?

This follows standard clinical practice, and best practice in modelling MDR-TB treatment from other literature in this field.

4. Authors considered Value TB dataset for economic analysis purpose. This dataset is based on figures from 78 health facilities and costs can significantly vary. Overall dataset is 7393 providers and only 564 for India. How do they assume representation with such a small number?

The Value TB dataset contains 213 unit cost observations from 18 health facilities in India, 269 observations from 28 health facilities in Philippines, and 280 observations from 21 facilities in Georgia. High quality cost data is very resource-intensive to collect, and the typical number of observations from a primary costing study is very small (1-5 health facilities). Value TB is the largest and most intensive study to estimate the unit costs of TB services to date, and the best possible current source for unit cost data in these settings. Further information summarizing typical methods and approaches of unit costing studies can be found here: Cunnama, Lucy, et al. "A systematic review of methodological variation in healthcare provider perspective tuberculosis costing papers conducted in low-and middle-income settings, using an Intervention-Standardised unit cost typology." Pharmacoeconomics 38.8 (2020): 819-837.

5. BPaL regimen is a standard therapy, there is also a likelihood of developing resistance and further amplification can happen. That will affect the treatment outcome as well as costing. How do authors explain this?

We agree with the reviewer that if BPaL-based regimens are widely adopted and frequently used inappropriately there is a chance of evolution of TB strains that are resistant to the new drugs, but representing this potential long-term impact was outside the capacity of our Markov model. We have added a sentence to our limitations section explaining this.

6. Instead of just focusing on new regimen, which will further develop resistance after a few years, can Whole Genome Sequencing add more value in accurate diagnosis and treatment in these countries, averting the costs, instead of suggesting a blind use of regimen?

Clinical use of whole genome sequencing is not an affordable option for most high-TB burden settings and is irrelevant to these trial results.

In my opinion, modelling studies have a major limitation that their assumptions are far away from the ground reality and so major policy decisions should be backed trials in the real world setting, then only economic analysis can be more accurate.

We agree that models and their results should always be represented as aids to decision making, rather than statements of scientific fact. However, models can be very helpful for translating trial results and aiding real-world decisions. The use of decision analytic models to represent the lifetime costs and effects of interventions is recommended as best practice in most guidelines for economic evaluation.

Reviewer #2: Publication is relevant and is well written.Comment are as follows
1. The Markov model is useful for making estimations for categorical time series. A practical model based on primary data would be the ideal analysis of the accrued benefits of making the switch to the newer Bpal regime.

We are unsure as to what the reviewer means by a ‘practical model’. We assume this is referring to a statistical analysis on individual-level data, rather than fitting the Markov model to aggregate data. Unfortunately, we did not have access to individual-level data for this analysis and so instead followed best practice in developing a population-level Markov model.

2. This is a 20 year cost benefit model. Did they make provisions for the effects of inflation?

The model is designed to represent the current value of future health states and costs. We therefore use a discount rate to represent time preferences and the declining purchasing power of currency over time.

3. Limitations of this study are as follows:-
a. This is a provider based CBA- a patient based model would offer very useful insight on the benefits of this new regime.

We completely agree, and we are working on a within-trial economic evaluation using primary unit cost data collected at trial sites from both the patient and provider perspectives. However, for this interim analysis for presented to the WHO Guidelines Decision Group, patient cost data was unavailable for the four countries included in this analysis. We recognize this as a limitation of the analysis in the Discussion section.

b. This are modelling based findings hinged on several assumptions.

We agree, and have noted this as a limitation.

Reviewer #3: This manuscript describes a modelled study of estimates of cost effectiveness of new regimens for the treatment of rifampicin/multi -drug resistant TB that contain bedaquiline, linezolid, pretomanid with or without moxifloxacin or clofazimine (BPaL, BPaLM, BPaLC) as used the TB-PRACTECAL trial. All the investigational regimens were found to be cost saving compared with the standard of care short or long regimens with BPaLM being the preferred regimen at willingness to pay per DALY averted of 0.5 DGD per capita. The paper is very well written and I only have very minor issues to raise.

1. On page the authors state that in 2020 there were 9.8 million TB infections in the world. This is erroneous. The authors are likely to be referring to people who developed active TB disease in that year as opposed to people who are infected with Mycobacterium tuberculosis , whose number is in billions ( about a third of the global population )

Thanks for catching this error; we’ve now revised the text here.

2. In table 1, the authors use the terms TB case detection rate while the currently preferred terms are Treatment Coverage because detection may not always lead to treatment .

Thanks; we’ve changed this wording throughout the paper.

Reviewer #4: Authors demonstrated that BPaL-based regimens were cost-effective in the treatment of RR-TB. The manuscript was well written with adequate information supporting the conclusion drawn. However, minor revision is required in this regard.

Abstract
Write TB in full on first mention

We’ve added this.

Introduction
1. Tuberculosis is the world’s second deadliest infection after COVID… I am not too sure about this statement. Please provide reference

We’ve added a reference here.

2. Add abbreviation to tuberculosis so that subsequence use of TB will be understood

We’ve added this.

3. Place RR-TB after …strain of TB that is resistant to rifampicin

We’ve moved this abbreviation from the end of the sentence.

4. It is quite strange you made statements without references. Provide references for statements beginning with

a. Tuberculosis is the world’s second deadliest…

We’ve added a reference here.

b. Each year about 470,000 people fall ill with a strain of TB that is resistant to rifampicin…

We’ve added a reference here.

c. Treatment options for RR-TB…

We’ve added a reference here.

d. Outcomes for RR-TB…

We’ve added a reference here.

Methods
1. Under Interventions and comparators, why were the study arms not presented consecutively?

This was for simplicity in communicating the drugs included in each arm, as the drug combination in arm 3 is also the backbone of arms 1 and 2.

2. Page 9, under cost, using laboratory in place of lab is appropriate. Why couldn’t you obtain the Value TB dataset for South Africa?

We’ve changed ‘lab’ to ‘laboratory’ here. The Value TB costing study was only conducted in five countries: Kenya, Ethiopia, Philippines, Georgia and India.

Results
1. Page 11, BPaLM would save $904-$80… why not $80-$904?
We’ve amended this.

Discussion
Page 12, please be consistent regarding SOC and SoC.

We’ve amended this.
