# Supplementary material for: Cost-effectiveness of short, oral treatment regimens for rifampicin resistant tuberculosis
Source: PLOS Glob Public Health. 2022 Dec 7;2(12):e0001337. doi: 10.1371/journal.pgph.0001337 (PMC10022130; doi:10.1371/journal.pgph.0001337)
Supplement: Peer review history

# Peer review: Decision Letter 0

**DOI:** 10.1371/journal.pgph.0001337.r001

**Authors:** Malaisamy Muniyandi

10 Oct 2022

PGPH-D-22-01344

Cost-effectiveness of short, oral treatment regimens for rifampicin resistant tuberculosis

PLOS Global Public Health

Dear Dr. Sweeney,

Thank you for submitting your manuscript to PLOS Global Public Health. After careful consideration, we feel that it has merit but does not fully meet PLOS Global Public Health’s publication criteria as it currently stands. Therefore, we invite you to submit a revised version of the manuscript that addresses the points raised during the review process.

Please submit your revised manuscript by Nov 09 2022 11:59PM. If you will need more time than this to complete your revisions, please reply to this message or contact the journal office at globalpubhealth@plos.org. When you're ready to submit your revision, log on to https://www.editorialmanager.com/pgph/ and select the 'Submissions Needing Revision' folder to locate your manuscript file.

Please include the following items when submitting your revised manuscript:

- A rebuttal letter that responds to each point raised by the editor and reviewer(s). You should upload this letter as a separate file labeled 'Response to Reviewers'.
- A marked-up copy of your manuscript that highlights changes made to the original version. You should upload this as a separate file labeled 'Revised Manuscript with Track Changes'.
- An unmarked version of your revised paper without tracked changes. You should upload this as a separate file labeled 'Manuscript'.

Guidelines for resubmitting your figure files are available below the reviewer comments at the end of this letter.

We look forward to receiving your revised manuscript.

Kind regards,

Malaisamy Muniyandi, Ph.D

Academic Editor

PLOS Global Public Health

Journal Requirements:

1. Please review your reference list to ensure that it is complete and correct. If you have cited papers that have been retracted, please include the rationale for doing so in the manuscript text, or remove these references and replace them with relevant current references. Any changes to the reference list should be mentioned in the rebuttal letter that accompanies your revised manuscript. If you need to cite a retracted article, indicate the article’s retracted status in the References list and also include a citation and full reference for the retraction notice.

2. Please send a completed 'Competing Interests' statement, including any COIs declared by your co-authors. If you have no competing interests to declare, please state "The authors have declared that no competing interests exist". Otherwise please declare all competing interests beginning with the statement "I have read the journal's policy and the authors of this manuscript have the following competing interests:"

3. Please amend your detailed Financial Disclosure statement. This is published with the article. It must therefore be completed in full sentences and contain the exact wording you wish to be published.

a. State the initials, alongside each funding source, of each author to receive each grant.

b. State what role the funders took in the study. If the funders had no role in your study, please state: “The funders had no role in study design, data collection and analysis, decision to publish, or preparation of the manuscript.”

If you did not receive any funding for this study, please simply state: “The authors received no specific funding for this work.”

4. We have noticed that you have uploaded Supporting Information files, but you have not included a list of legends. Please add a full list of legends for your Supporting Information files after the references list. 

Additional Editor Comments (if provided):

Manuscript number PONE-D-22-20832

Title: Prevention of relapse in Drug Sensitive Pulmonary Tuberculosis patients with and without Vitamin D3 supplementation: A double blinded randomized control clinical trial

Overall comments: This manuscript focused on an important topic to study the role of vitamin D supplementation in patients with active pulmonary tuberculosis in the Indian population to sputum smear and culture conversion as well as the prevention of relapse through double blinded randomised control clinical trial. Overall this research address important issue and well written manuscript. I recommended for publication with minor revisions.

Specific comments

• Abstract: under methods Revised National Tuberculosis Control Program can be changed as a National TB Elimination Programme or if the name of the guideline is Revised National Tuberculosis Control Program, it can be retain.

• The full document the term ‘relapse’ to be changed as a ‘recurrence’ and the term ‘subject’ to be changed as a ‘participants’

• In the background, death due to TB in 2020 is given; authors can update the reference to 2022, if it is available

• Abbreviations to be given first time and then used subsequently; Example: PTB; MDR-TB; LPA; BMI; ATT

• Line 106; the sentence starts with number 2; can be changed to text (ie., two)

• Authors can provide Clinical Trial Registry member and Ethics Number.

• Any quality control mechanism followed in the laboratory investigations and data collection; if it is done, that need to be added under methodology section

• Since it is a randomized double-blind placebo-controlled trial done in three different sites; methodology need to be given with more clarity with subtitles like study design, study area, study population, study procedures, definitions used, data collection and data management (including data entry, cleaning, analysis and statistical tools used) and ethical issues.

• Similarly, results section there is a need to provide subtitles like demographic profile of study population, clinical profile, clinical status at baseline and at the end of follow-up; association with clinical outcomes and salient findings (like smear and culture conversion, time to conversion; recurrent rate and factors associated with it). Authors collected large volume of data, but it seems to be not done deep and detailed analysis. If possible authors can try and provide detailed analysis.

• It was mentioned that final analysis was done those completed 6-month treatment; whether they have cured or only completed.

• Why recurrent rate was high among males; is there any explanation for this

• Adverse events (14 patients) need to explained

• Overall discussion part need to strengthen; why authors compared this results with LTBI infection; there is no explanation for controversial results like low vitamin D Levels with increased risk of PTB. The discussion may be low vitamin D levels were associated with an increased risk for progression to tuberculosis, but not recurrence. There are evidences on vitamin D supplementation is likely to have a primary preventive effect on the incidence risk as well as a beneficial effect on the anti-tuberculosis treatment. There is a need to highlight that no association with recurrence of TB and vitamin D supplementation.

[Note: HTML markup is below. Please do not edit.]

Reviewers' comments:

Reviewer's Responses to Questions

**Comments to the Author**

1. Does this manuscript meet PLOS Global Public Health’s publication criteria? Is the manuscript technically sound, and do the data support the conclusions? The manuscript must describe methodologically and ethically rigorous research with conclusions that are appropriately drawn based on the data presented.

Reviewer #1: Yes

Reviewer #2: Yes

Reviewer #3: Yes

Reviewer #4: Yes

**********

2. Has the statistical analysis been performed appropriately and rigorously?

Reviewer #1: I don't know

Reviewer #2: Yes

Reviewer #3: I don't know

Reviewer #4: N/A

**********

3. Have the authors made all data underlying the findings in their manuscript fully available (please refer to the Data Availability Statement at the start of the manuscript PDF file)?

The PLOS Data policy requires authors to make all data underlying the findings described in their manuscript fully available without restriction, with rare exception. The data should be provided as part of the manuscript or its supporting information, or deposited to a public repository. For example, in addition to summary statistics, the data points behind means, medians and variance measures should be available. If there are restrictions on publicly sharing data—e.g. participant privacy or use of data from a third party—those must be specified.

Reviewer #1: Yes

Reviewer #2: Yes

Reviewer #3: Yes

Reviewer #4: Yes

**********

4. Is the manuscript presented in an intelligible fashion and written in standard English?

PLOS Global Public Health does not copyedit accepted manuscripts, so the language in submitted articles must be clear, correct, and unambiguous. Any typographical or grammatical errors should be corrected at revision, so please note any specific errors here.

Reviewer #1: Yes

Reviewer #2: Yes

Reviewer #3: Yes

Reviewer #4: Yes

**********

5. Review Comments to the Author

Please use the space provided to explain your answers to the questions above. You may also include additional comments for the author, including concerns about dual publication, research ethics, or publication ethics. (Please upload your review as an attachment if it exceeds 20,000 characters)

Reviewer #1: This modelling based study indicates that BPaL-based regimens are likely to be cost-saving and more effective than the current standard of care in a range of settings and it suggests that countries should consider programmatic uptake of BPaL-based regimens. The study has many important implications especially in the context of poor success rates with the current regimen used for treating drug resistant TB forms such as MDR and XDR-TB and increasing levels of drug resistance in these study countries.

Following are some points to consider

1. At model entry, mean age was assumed to be 35 (Supplementary Table 2): Can authors provide justification for this number?

2. At the end of each month, patients could transition to the next month of treatment, loss to follow-up, treatment failure, or death: I am unable to understand how they can transition to failure just in next month? On what basis?

3. Following the failure of initial regimen, authors assume that patients are put on long SOC rescue regimen regardless of their initial regimen. What is the basis for this assumption?

4. Authors considered Value TB dataset for economic analysis purpose. This dataset is based on figures from 78 health facilities and costs can significantly vary. Overall dataset is 7393 providers and only 564 for India. How do they assume representation with such a small number?

5. BPaL regimen is a standard therapy, there is also a likelihood of developing resistance and further amplification can happen. That will affect the treatment outcome as well as costing. How do authors explain this?

6. Instead of just focusing on new regimen, which will further develop resistance after a few years, can Whole Genome Sequencing add more value in accurate diagnosis and treatment in these countries, averting the costs, instead of suggesting a blind use of regimen?

In my opinion, modelling studies have a major limitation that their assumptions are far away from the ground reality and so major policy decisions should be backed trials in the real world setting, then only economic analysis can be more accurate.

Reviewer #2: Publication is relevant and is well written.Comment are as follows

1. The Markov model is useful for making estimations for categorical time series. A practical model based on primary data would be the ideal analysis of the accrued benefits of making the switch to the newer Bpal regime.

2. This is a 20 year cost benefit model. Did they make provisions for the effects of inflation?

3. Limitations of this study are as follows:-

a. This is a provider based CBA- a patient based model would offer very useful insight on the benefits of this new regime.

b. This are modelling based findings hinged on several assumptions.

Reviewer #3: This manuscript describes a modelled study of estimates of cost effectiveness of new regimens for the treatment of rifampicin/multi -drug resistant TB that contain bedaquiline, linezolid, pretomanid with or without moxifloxacin or clofazimine (BPaL, BPaLM, BPaLC) as used the TB-PRACTECAL trial. All the investigational regimens were found to be cost saving compared with the standard of care short or long regimens with BPaLM being the preferred regimen at willingness to pay per DALY averted of 0.5 DGD per capita. The paper is very well written and I only have very minor issues to raise.

1. On page the authors state that in 2020 there were 9.8 million TB infections in the world. This is erroneous. The authors are likely to be referring to people who developed active TB disease in that year as opposed to people who are infected with Mycobacterium tuberculosis , whose number is in billions ( about a third of the global population )

2. In table 1, the authors use the terms TB case detection rate while the currently preferred terms are Treatment Coverage because detection may not always lead to treatment .

I am not skilled enough to comment on the statistical analysis used in this paper

Reviewer #4: Authors demonstrated that BPaL-based regimens were cost-effective in the treatment of RR-TB. The manuscript was well written with adequate information supporting the conclusion drawn. However, minor revision is required in this regard.

Abstract

Write TB in full on first mention

Introduction

1. Tuberculosis is the world’s second deadliest infection after COVID… I am not too sure about this statement. Please provide reference

2. Add abbreviation to tuberculosis so that subsequence use of TB will be understood

3. Place RR-TB after …strain of TB that is resistant to rifampicin

4. It is quite strange you made statements without references. Provide references for statements beginning with

a. Tuberculosis is the world’s second deadliest…

b. Each year about 470,000 people fall ill with a strain of TB that is resistant to rifampicin…

c. Treatment options for RR-TB…

d. Outcomes for RR-TB…

Methods

1. Under Interventions and comparators, why were the study arms not presented consecutively?

2. Page 9, under cost, using laboratory in place of lab is appropriate. Why couldn’t you obtain the Value TB dataset for South Africa?

Results

1. Page 11, BPaLM would save $904-$80… why not $80-$904?

Discussion

Page 12, please be consistent regarding SOC and SoC.

**********

6. PLOS authors have the option to publish the peer review history of their article (what does this mean?). If published, this will include your full peer review and any attached files.

**Do you want your identity to be public for this peer review?** If you choose “no”, your identity will remain anonymous but your review may still be made public.

For information about this choice, including consent withdrawal, please see our Privacy Policy.

Reviewer #1: No

Reviewer #2: **Yes: **Dr Lydia Atambo

Reviewer #3: No

Reviewer #4: No

**********

[NOTE: If reviewer comments were submitted as an attachment file, they will be attached to this email and accessible via the submission site. Please log into your account, locate the manuscript record, and check for the action link "View Attachments". If this link does not appear, there are no attachment files.]

While revising your submission, please upload your figure files to the Preflight Analysis and Conversion Engine (PACE) digital diagnostic tool, https://pacev2.apexcovantage.com/. PACE helps ensure that figures meet PLOS requirements. To use PACE, you must first register as a user. Registration is free. Then, login and navigate to the UPLOAD tab, where you will find detailed instructions on how to use the tool. If you encounter any issues or have any questions when using PACE, please email PLOS at figures@plos.org. Please note that Supporting Information files do not need this step.

# Author comment: Author response to Decision Letter 0

**DOI:** 10.1371/journal.pgph.0001337.r002

8 Nov 2022

Attachment Submitted filename: PRACTECAL CEA_Response to reviewers.docx Click here for additional data file.

# Editor report: Decision Letter 1

**DOI:** 10.1371/journal.pgph.0001337.r003

**Authors:** Malaisamy Muniyandi

10 Nov 2022

Cost-effectiveness of short, oral treatment regimens for rifampicin resistant tuberculosis

PGPH-D-22-01344R1

Dear Dr Sedona

We are pleased to inform you that your manuscript 'Cost-effectiveness of short, oral treatment regimens for rifampicin resistant tuberculosis' has been provisionally accepted for publication in PLOS Global Public Health.

Before your manuscript can be formally accepted you will need to complete some formatting changes, which you will receive in a follow up email. A member of our team will be in touch with a set of requests.

Please note that your manuscript will not be scheduled for publication until you have made the required changes, so a swift response is appreciated.

IMPORTANT: The editorial review process is now complete. PLOS will only permit corrections to spelling, formatting or significant scientific errors from this point onwards. Requests for major changes, or any which affect the scientific understanding of your work, will cause delays to the publication date of your manuscript.

If your institution or institutions have a press office, please notify them about your upcoming paper to help maximize its impact. If they'll be preparing press materials, please inform our press team as soon as possible -- no later than 48 hours after receiving the formal acceptance. Your manuscript will remain under strict press embargo until 2 pm Eastern Time on the date of publication. For more information, please contact globalpubhealth@plos.org.

Thank you again for supporting Open Access publishing; we are looking forward to publishing your work in PLOS Global Public Health.

Best regards,

Malaisamy Muniyandi, Ph.D

Academic Editor

PLOS Global Public Health

***********************************************************

Reviewer Comments (if any, and for reference):
